# Method to analyse welfare effect of transferable quotas in an open economy with rent-seeking for quotas

**DOI:** 10.1016/j.mex.2022.101722

**Published:** 2022-05-05

**Authors:** Yoshihiro Hamaguchi

**Affiliations:** Department of Management Information, Kyoto College of Economics

**Keywords:** ITQ, Lobbying effort, Opening-up trade, Renewable resource

## Abstract

Governments have introduced local individual transferable quotas (ITQs) to maintain fishery resource sustainability. However, quota rent-seeking activities by fishermen may weaken ITQ's resource conservation effect in countries with immature private property systems. This method is developed to analyse how opening-up trade impacts resource management and welfare under quota rent-seeking activities by applying rent-seeking for emission permits to rent-seeking for ITQ in a two-country model to investigate the policy effect of opening-up trade on renewable resources. The method helps analyse ITQ's welfare effect in an open economy with quota rent-seeking activities and the welfare effect of opening-up trade via quota lobbying, comparing an autarky with an open economy. Quota changes in the foreign country specialising in fishery production influence quota prices in both the foreign country and the home country, diversifying away from fishery to manufacturing production, thereby affecting fishery and manufacturing goods production in the home country. Thus, fishery policy indirectly affects welfare in the home country. The method can be applied to investigate fisheries disputes under international resource management. In sum, this method facilitates:

• Analysing the welfare effect of quotas with quota lobbying in a two-country model;
• Examining the policy effect of opening-up trade on quota rent-seeking and fisheries production, comparing an autarky with an open economy; and
• Investigating fisheries disputes under the international management of fishery resources.

## Graphical abstract

**Figure:**
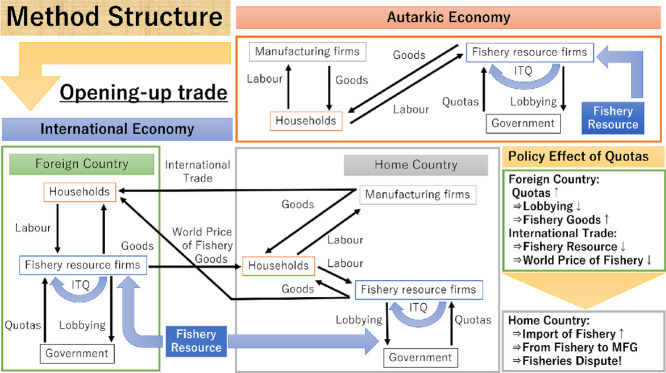

Specifications table

**Table:** 

Subject Area:	
More specific subject area:	*Environmental Economics, Resource Economics*
Method name:	*Static general equilibrium analysis*
Name and reference of original method:	*Rode, Ashwin. 2021. “Rent-Seeking over Tradable Emission Permits: Theory and Evidence.'' Environmental Resource and Economics 78(2): 257–285.*
	*Takarada, Yasuhiro, Weijia Dong, and Takeshi Ogawa. 2013. “Shared Renewable Resources: Gains from Trade and Trade Policy.” Review International Economics 21(5): 1032–1047.*
Resource availability:	*None*

## Method details

This method is developed by applying the policy effect of opening-up trade on renewable resources by Takarada et al. [21] to rent-seeking for emission permits by Rode [19]. In the present model, I consider fishery resources as renewable resources and replace rent-seeking for emission permits with rent-seeking for individual transferable quotas (ITQs). This model setting enables investigating how opening-up trade influences fishery resources and rent-seeking activities by comparing an autarkic economy with an open economy. This model analysis has already been published by Hamaguchi [12]. However, I believe that its analytical approach is versatile and I describe its main points in this journal, which is open access. I analyse a two-country model in which the home country and the foreign country (whose variables are denoted by an asterisk) trade mutually. This method focuses on short-term analysis (for further description of the method, please refer to Hamaguchi [12]).

### Short literature review

As fisheries resources are typically common, lack of private ownership leads to overexploitation of these resources. This resource depletion problem is known as the tragedy of the commons. A policy instrument to prevent this is the individual transferable quotas (ITQ). The factors that cause this tragedy and the measures to prevent it are briefly summarised by Libecap [16]. It is said that if externality can be internalised by introducing ITQs to fisheries resources, resource depletion can be avoided. However, this policy has long been debated as it creates rents associated with the allocation of quotas (e.g. [1,7]). More recently, there has been concern that rent-seeking in fisheries may undermine the sustainability of fisheries ([13,20]).

Theoretical analyses point out that, because of open access, the rent-seeking involved in ITQs undermines the efficiency of resource management [2,5]. Several analyses indicate that grandfathered quotas lead to allocated rents and consequently undermine the efficiency of the ITQ system [10,11]. Rent-seeking related to scallop fisheries in the Atlantic Ocean in North America is analysed in detail in [6]. After touching on the background of fisheries management and rent-seeking in this area, the study uses a simple estimation equation to estimate the marginal and average profit of the fishing effort. The study by Whitmarsh [23] describes rent-seeking associated with cost-reducing new technologies. Moreover, Foley et al. [9] detail how policy and social relations lead to rent-seeking through a case study analysis of the Canadian shrimp fishery and several solutions are proposed from the Institutional Analysis and Development (IAD) analytical framework by Imperial and Tracy [15].

However, to come up with effective measures to prevent rent-seeking involving ITQs, it is necessary to identify the factors involved. Empirical studies indicate several factors (e.g. [8,14,17]). The theoretical analysis by Boyce [4] reveals that governments do not choose rent-generating policy instruments when the welfare weight for some economic agent is high. In addition, not only do rent gains depend on past rates of resource exploitation [3], but the size of the rent also depends on the extent of rent dissipation prior to the introduction of the ITQ [18].

Here, globalisation is expected to have a major impact on rent-seeking. For example, with the development of free trade, developing countries will try to increase exports of seafood products. However, ITQ catch limits will hinder the expansion of exports. Fisherers will, therefore, actively lobby governments to increase their catches. As a result, they would obtain catch quotas above the regulatory level as rents. Illegally expanded ITQs would lead to a decline in fishing stocks. Here, free trade in fisheries products will cause further resource depletion. The share of foreign fisheries products is increasing in developing countries' export destinations. Imports of cheap foreign seafood would depress domestic seafood prices in exporting countries and reduce the profits of fishermen. As a countermeasure, fishermen in exporting countries appeal to their governments to increase their catch quotas. If their governments were to accept this political demand, a global fisheries depletion crisis would occur.

In a global economy where effects through trade exist, one must consider the economies and policies of foreign countries as well as one's own to conserve fisheries resources. The research methodology developed by this study can analyse the impact of globalisation on rents related to fisheries resources and ITQs. The results of a detailed analysis by Hamaguchi [12] point to the risk of undesirable consequences of free trade, as to whether it improves the welfare of a country depends on the political situation in the foreign country. It therefore states that co-ordinated resource management policies between states are essential to promote global resource management. This short review will complement the research context of Hamaguchi [12].

### Consumption and production

The population in the home country is represented by L, and households cannot move between countries. Households supply their labour to firms in the resource and manufacturing goods sectors. Firms in the resource goods sector harvest renewable fishery resources and those in the manufacturing sector produce manufacturing goods. The utility function of households is denoted by the following logarithmic function of the Cobb-Douglas formation:

(1) u=βlogH+(1−β)logM,

where H and M represent the consumption of resource and manufacturing goods, respectively, and β is the taste parameter of consumption. Here, $\beta =\beta^{*}$ is assumed to simplify the analysis. The budget constraint of the households is represented as $pH_{D}+M_{D}=I$, where p is the relative resource goods price and I is households’ total income. The households choose the consumption of resource and manufacturing goods to maximise their utility in (1) subject to the budget constraint. This yields the demand functions of resource and manufactured goods as $H_{D}=\beta I/p$ and $M_{D}=\left(1-\beta \right)I$, respectively.

Firms in the manufacturing goods sector produce homogenous goods by employing a unit of labour in a perfect competitive market. This leads to $M_{P}=L_{M}$, where $L_{M}$ is the labour input in the manufacturing sector. Firms in the resource goods sector harvest renewable fishery resources by employing a unit of labour. The production function is expressed as follows:

(2) $$h\left(i\right)=STl_{H,i},$$

where h(i) and $l_{H,i}$ denote firm *i*'s harvesting resource goods and labour input, respectively, S is the exogenous renewable fisheries resource, and T represents the length of the fishing season. Aggregating (2) yields the following aggregated production function:

(3) $$\int_{i=0}^{f}h\left(i\right)di=H_{P}=TSL_{H},$$

where $L_{H}$ is the aggregated labour input and f denotes the number of firms in the sector.

### Quota rent-seeking

The country-specific ITQ market prevails in competition. The government sets a total quota of $\bar{H}$ in the domestic ITQ market and distributes the grandfathering quota $\bar{h}$ to f firms in the resource goods sector. This indicates that $\int_{i=0}^{f}\bar{h}\left(i\right)di=f\bar{h}\left(i\right)=\bar{H}$ must hold. These firms can influence the quota allocation process via lobbying efforts, which is regarded as a rent-seeking activity. The allocation process is described as follows: First, through the announcement of the government, each firm knows an initial allocation of $\bar{h}\left(i\right)$, which represents firm i’s initial allocation. Each firm with knowledge of the allocation attempts to contest the quotas. This results in the government redistributing a post-contest quota allocation of $\tilde{h}\left(i\right)$ to firms with lobbying efforts. As in Tullock [22], the post-contest allocation of firm i is represented by the following contest function:

(4) $$\tilde{h}\left(i\right)=\left\{ \begin{array}{c} \begin{array}{c} a\bar{h}\left(i\right)+\psi \left(\frac{l_{X,i}}{\int_{i=0}^{f}l_{X,i}di}\right),\;\;\;\;if\;\;\;\;\frac{l_{X,i}}{\int_{i=0}^{f}l_{X,i}di}>0,\;\\ \bar{h}\left(i\right),\;\;\;\;\;\;\;\;\;\;\;\;\;\;\;\;\;\;\;\;\;\;\;\;\;\;\;\;\;\;\;\;\;\;\;\;\;\;\;\;\;otherwise,\;\;\;\;\;\;\;\;\;\;\;\;\;\;\;\;\; \end{array}\;\;\;\;\;\; \end{array}\right.$$

where $l_{X.i}$ denotes firm i’s lobbying effort and $\psi \equiv \bar{H}-\int_{i=0}^{f}a\bar{h}\left(i\right)di=\bar{H}-af\bar{h}\left(i\right)=\left(1-a\right)\bar{H}$ represents the number of quotas under the contest pressure. Aggregating $l_{X.i}$, the total lobbying effort is represented as $\int_{j=0}^{f}l_{X,j}dj=fl_{X,j}=L_{X}$.

The contest function in (4) signifies that a firm's quota level expands based on the ratio of that firm's lobbying effort to all firms' total lobbying efforts. Parameter a, which is commonly known by firms and the government, suggests that firms are managed identically by the government under the redistribution process. Here, parameter a determines $\psi \in [0,\;\bar{H}]$ in the present model, which leads to a positive contested quota level.

On realising the allocation of the quotas via the contest in (4), firm i maximises the profit, taking as given the lobbying efforts of the other firms, as follows:

(5) $$\max_{h\left(i\right)>0,\;l_{X,i}\geq 0}\begin{array}{c} \pi =ph\left(i\right)-wl_{H,i}-wl_{X,i}-r\left[h\left(i\right)-\tilde{h}\left(i\right)\right]=\left[ph\left(i\right)-wl_{H,i}\right]-rh\left(i\right)+\left[r\tilde{h}\left(i\right)-wl_{X,t}\right],\;\; \end{array}$$

where p is the harvest goods price, w is the wage rate, and r is the quota price. Firm i's choice of lobbying effort is represented as $\left[r\tilde{h}\left(i\right)-wl_{X,t}\right]$ in (5) and regarded as detrimental to society in the present model. The zero-profit condition of the harvest and the first-order condition of the lobbying efforts are, respectively represented as follows:

(6) $$p=\frac{w}{ST}+r,$$

(7) $$r\psi \left[\frac{l_{X,-i}}{{\left(l_{X,i}+l_{X,-i}\right)}^{2}}\right]=w,\;$$

where $l_{X,-i}$ denotes the lobbying efforts of all aggregated firms except for firm i.

Here, the lobbying effort in equilibrium is derived (see Rode [19] for the rigorous proof). Using (7), the best-response function is expressed as follows:

(8) $$l_{X,i}=\left\{ \begin{array}{c} \begin{array}{c} \sqrt{\frac{r\psi l_{X,-i}}{w}}-l_{X,\;-i},\;\;\;\;\;\;if\;\;\;\;l_{X,-i}\in \left(0,\frac{r\psi }{w}\right)>0,\\ 0,\;\;\;\;\;\;\;\;\;\;\;\;\;\;\;\;\;\;\;\;\;\;\;\;\;\;\;\;\;\;\;\;if\;\;\;\;l_{X,i}\geq \frac{r\psi }{w},\;\;\;\;\;\;\;\;\;\;\;\;\;\;\;\;\;\; \end{array}\; \end{array}\right.$$

where a Nash equilibrium is regarded as a strategy profile of all firms engaging in lobbying. The first-order condition of the lobbying effort in (7) is held for i=0,1,2,⋯,f under all f firms making positive lobbying efforts. Aggregating (7) over all i, the lobbying effort is represented as follows:

(9) $$l_{X}=\frac{r\psi \left(f-1\right)}{\int_{i=0}^{f}wdi},\;\;\;for\;\;\;\;i=0,\;1,\;2,\;\cdots ,\;f,$$

where $l_{X}=l_{X,i}$ for all i holds because symmetric equilibrium is assumed for all firms. Under $l_{X,\;i}>0$ for i=1,2,⋯,f in a symmetric equilibrium, the best-response function in (9) can be rewritten as follows:

(10) $$l_{X}=\sqrt{\frac{r\psi l_{X,-i}}{w}},\;\;\;\;for\;\;\;\;i=0,\;1,\;2,\;\cdots ,\;f.$$

Combining (9) with (10) yields the following:

(11) $$l_{X,-i}=\frac{wr\psi {\left(f-1\right)}^{2}}{{\left(\int_{i=0}^{f}wdi\right)}^{2}},\;\;\;\;for\;\;\;\;i=0,\;1,\;2,\;\cdots ,\;f,\;$$

which is further rewritten as follows:

(12) $$l_{X,i}=\frac{wr\psi {\left(f-1\right)}^{2}}{{\left(\int_{i=0}^{f}wdi\right)}^{2}}\left[\int_{i=0}^{f}wdi-w\left(f-1\right)\right],\;\;\;\;for\;\;\;\;i=0,\;1,\;2,\;\cdots ,\;f,$$

which denotes firm i's optimal lobbying effort level in equilibrium. Here, all firms face the same lobbying cost under symmetric equilibrium. Thus, all firms can engage in lobbying only when $\int_{i=0}^{f}wdi/\left(f-1\right)>w$ for i=0,1,2,⋯,f holds. As such, rewriting (12) yields the following lobbying effort level per firm and total lobbying effort level under a symmetric equilibrium, respectively:

(13) $$l_{X}=\frac{r\psi \left(f-1\right)}{wf^{2}},\;\;\;\;\;\;\;\;L_{X}=\frac{r\psi \left(f-1\right)}{f},$$

where the lobbying effort becomes identical among firms.

Here, it may seem that this rent-seeking on ITQs is fraught with collective action problems. This is because the benefits of the expanded ITQs resulting from rent-seeking will go to all firms in the industry. This will create firms that seek to benefit from the expansion of ITQs without any lobbying efforts at all. In other words, a free-riding problem arises. In this modelling analysis, it is implicitly assumed that this free-riding does not occur.

The labour market-clearing condition is represented as $L_{H}+L_{M}+L_{X}=L$, where L is the labour endowment. In the present model, the manufacturing good is regarded as the numeraire, and thus, the price of the manufacturing good becomes one. The wage rate in the manufacturing goods sector becomes one based on the manufacturing firm's profit maximisation problem. The wage rate equalises among sectors, and thus, the wage rate in equilibrium leads to w=1.

### Autarkic economy equilibrium

In an autarkic economy without international trade, firms in the resource goods sector harvest resource goods and those in the manufacturing goods sector produce manufacturing goods in each country. This production structure implies $w=w^{*}=1$. The total income of households in each country is represented as $I_{A}=L$ and $I_{A}^{*}=L^{*}$. The goods market-clearing condition indicates that the domestic demands for resource and manufacturing goods equalise the domestic supply of these goods.

Using (3), (6), (8), $H_{D}$,$M_{D}$,$I_{A}=L$, and $L_{H}+L_{M}+L_{X}=L$ yields the following labour conditions:

(13) $$L_{H,A}=\frac{\beta \left(L-\hat{f}r_{A}\right)}{1+ST\left(1-\beta \right)r_{A}},$$

(14) $$L_{M,A}=\left(1-\beta \right)\left(L-\hat{f}r_{A}+\frac{\beta \left(L-\hat{f}r_{A}\right)}{1+ST\left(1-\beta \right)r_{A}}\right),$$

(15) $$L_{X,A}=\hat{f}r_{A},$$

$$\hat{f}\equiv \left(f-1\right)\left(1-a\right)\bar{h},$$

where (13) and (14) indicate the volume of employment in the resource and manufacturing goods sectors, respectively, and (15) denotes the volume of the lobbying effort in the quota contest. Note that $\hat{f}$ represents the post-contest allocated quota.

The quota market-clearing condition in an autarkic economy is represented as follows:

(16) $$fr_{A}\left[\tilde{h}\left(i\right)-h\left(i\right)\right]=0\;\;\;\Leftrightarrow \;\;\;\;h\left(i\right)=\frac{\bar{H}}{f}=\bar{h},\;$$

which implies that the quota distributed to firm i leads to an average quota in equilibrium. Combining $H_{P}=TSL_{H}$ with (12) yields the following volume of employment in the resource goods sector in equilibrium:

(17) $$L_{H}=\frac{f\bar{h}}{ST},$$

where (13) and (17) are used to derive the following quota price in equilibrium:

(18) $$r_{A}=\frac{\left(\beta LST/\bar{h}\right)-f}{ST\left[f\left(1-\beta \right)+\beta \left(f-1\right)\left(1-a\right)\right]}.$$

Finally, $u_{A}=\beta \log\beta +\left(1-\beta \right)\log\left(1-\beta \right)+\log I_{A}-\beta \log p_{A}$ is derived by substituting $H_{D}=\beta I/p$ and $M_{D}=\left(1-\beta \right)I$ into (1). Using (11), (13), $I_{A}=L_{A}$, and $H_{P}=TSL_{H}$, the rewritten utility function can be represented as follows:

(19) $$u_{A}=\left(1-\beta \right)\log\left(1-\beta \right)+\beta \log\left(f\bar{h}\right)+\left(1-\beta \right)\log\left[L+\bar{h}r_{A}\left\{1+a\left(f-1\right)\right\}\right],$$

which implies that welfare changes according to the endogenous quota price, the distributed quota level, and the total number of resource goods firms.

### Open economy equilibrium

To simplify the analysis of the present model, we focus on the following production pattern as demonstrated by Rode [19]: the home country has a diversified resource and manufacturing goods production structure, while the foreign country has a specialised resource goods production structure. This implies that w=1 and $w^{*}\geq 1$. Using (6), we derive $p_{A}>p_{A}^{*}$, which is rewritten as $r_{A}>r_{A}^{*}$. This inequality must hold under the following parameter conditions:

$$f^{*}>f,\;\;\;\;{\bar{h}}^{*}>\bar{h},\;\;\;\;\frac{{\bar{h}}^{*}}{\bar{h}}>\frac{f\left(f^{*}-1\right)}{f^{*}\left(f-1\right)},$$

which guarantee that quotas distributed to firms in the foreign country are larger than in the home country, and rent-seeking activities in the foreign country are more active than in the home country. Additionally, the parameter conditions guarantee a comparative advantage of the foreign country in the production of resource goods.

Using w=1 and $w^{*}\geq 1$ in (6) yields the following world price of resource goods after the trade:

(20) $$p_{T}=\frac{1}{ST}+r_{T}=\frac{w^{*}}{ST}+r_{T}^{*},$$

where the marginal product of resource goods in the home country is equal to that in the foreign country via the world price after the trade. Domestic firms in the resource goods sector produce resource goods via the production function of $H_{P}=STL_{H}$ and foreign firms in the resource goods sector produce resource goods via the production function of $H_{P}^{*}=ST\left(L-L_{X}^{*}\right)$. Combining $L_{XA}=\hat{f}r_{A}$ with (15) yields $L_{X}^{*}={\hat{f}}^{*}r_{T}^{*}$ and ${\hat{f}}^{*}\equiv \left(f^{*}-1\right)\left(1-a\right){\bar{h}}^{*}$ in the open economy. Furthermore, $I_{A}=L$ and $I_{A}^{*}=L^{*}$ can be rewritten as follows:

(21) $$I_{T}=L-\hat{f}r_{T}+STL_{HT}r_{T},\;\;\;\;I_{T}^{*}=p_{T}ST\left(L-{\hat{f}}^{*}r_{T}^{*}\right),$$

which indicate the total income of the home and foreign countries after the trade, respectively. The labour market-clearing condition in the foreign country is represented as follows:

(22) $$L_{XT}^{*}=\beta L^{*}-{\hat{f}}^{*}.$$

Substituting (21) and (22) for $H_{D}=\beta I_{T}/p_{T}$ yields the following demand for resource goods in each country:

(23) $$H_{DT}=\frac{\beta L}{p_{T}}\left[L+r_{T}\left(STL_{HT}-\hat{f}\right)\right],\;\;\;\;H_{DT}^{*}=\beta ST\left(L-{\hat{f}}^{*}r_{T}^{*}\right).$$

Here, the market-clearing condition for resource goods is represented as $H_{P}+H_{P}^{*}=H_{DT}+H_{DT}^{*}$. This equation can be rewritten using (20) and (22) as follows:

(24) $$L_{HT}=\frac{\beta \left(L-\hat{f}r_{T}\right)-ST\left(1-\beta \right)\left(L-{\hat{f}}^{*}r_{T}^{*}\right)\left(1/ST+r_{T}\right)}{1+STr_{T}\left(1-\beta \right)},$$

which signifies the volume of employment in the resource goods sector under an open economy. Note that $0<L_{HT}<L$ must hold in (24) to guarantee that the home country diversifies the production of the resource and manufacturing goods after the trade. The inequality of $L_{HA}>L_{H}$ derived using (13) and (24) leads to $0<L_{HT}<L$, which is further rewritten as follows:

$$L>L_{HT}\;\;\;\Rightarrow \;\;\;\;ST\left(1-\beta \right)\left(L-{\hat{f}}^{*}r_{T}^{*}\right)\left(1/ST+r_{T}\right)+L\left(1-\beta \right)+\beta \hat{f}r_{T}+STL\left(1-\beta \right)r_{T}>0,$$

where the inequality of $0<L_{HT}<L$ holds in the present model.

Combining (17) with (24) yields the following quota price in the home country:

(25) $$r_{T}=\frac{\left(2\beta -1\right)L+\left(1-\beta \right){\hat{f}}^{*}r_{T}^{*}-f\bar{h}/ST}{f\bar{h}\left(1-\beta \right)+\beta \hat{f}+ST\left(1-\beta \right)\left(L-{\hat{f}}^{*}r_{T}^{*}\right)},$$

and substituting (22) and (23) for $H_{P}^{*}=ST\left(L-L_{X}^{*}\right)$ yields the following quota price in the foreign country:

(26) $$r_{T}^{*}=\frac{STL/{\bar{h}}^{*}-f^{*}}{ST\left(f^{*}-1\right)\left(1-a\right)}.$$

Finally, $u_{T}=\beta \log\beta +\left(1-\beta \right)\log\left(1-\beta \right)-\beta \log p_{T}+\log I_{T}$ is derived by substituting $H_{D}=\beta I_{T}/p_{T}$ and $p_{T}H_{D}+M_{D}=I_{T}$ into (1). Using (17), (20)–(23), and $H_{D}=\beta I_{T}/p_{T}$, the utility function can be rewritten as follows:

(27) $$u_{T}=\left(1-\beta \right)\log\left(1-\beta \right)+\beta \log\left(f\bar{h}\right)+\left(1-\beta \right)\log\left[L+\bar{h}r_{T}\left\{1+a(f-1)\right\}\right],$$

(28) $$u_{T}^{*}=\beta \log\beta +\beta \log\left(1-\beta \right)+\left(1-\beta \right)\log\left(\frac{1}{ST}+r_{T}\right)+\log\left(L-{\hat{f}}^{*}r_{T}^{*}\right)+\log\left(ST\right),$$

where (27) and (28) denote the welfare in the home and foreign country, respectively. Note that welfare in the home country changes according to its quota price, while welfare in the foreign country changes according to its own and the home country's quota prices.

### Robustness check

Finally, we mention the assumptions of the model. We assume that β between two countries is the same. It may feel that the assumptions are strong. Here, we suppose $\beta \neq \beta^{*}$. Then, the labour market-clearing condition in the foreign country in (22) is rewritten as $L_{XT}^{*}=\beta^{*}L^{*}-{\hat{f}}^{*}$. Similarly, the demand for resource goods in foreign country in (23), and the volume of employment in the resource goods sector under an open economy in (24) are rewritten, respectively as follows:

$$H_{DT}^{*}=\beta^{*}ST\left(L-{\hat{f}}^{*}r_{T}^{*}\right),$$

$$L_{HT}=\frac{\beta \left(L-\hat{f}r_{T}\right)-ST\left(1-\beta^{*}\right)\left(L-{\hat{f}}^{*}r_{T}^{*}\right)\left(1/ST+r_{T}\right)}{1+STr_{T}\left(1-\beta \right)},$$

the two equations are further used to rewrite the quota price in the home country in (25) as follows:

$$r_{T}=\frac{\left(\beta +\beta^{*}-1\right)L+\left(1-\beta^{*}\right){\hat{f}}^{*}r_{T}^{*}-f\bar{h}/ST}{f\bar{h}\left(1-\beta \right)+\beta \hat{f}+ST\left(1-\beta^{*}\right)\left(L-{\hat{f}}^{*}r_{T}^{*}\right)}.$$

Finally, β in the foreign utility function is also replaced by $\beta^{*}$, but there is no change other than these equations.

Here, a comparative statics analysis of $\beta^{*}$ is performed. Since $\beta^{*}$ does not affect foreign quota prices, an increase in $\beta^{*}$ monotonically increases the demand for foreign resource goods. However, the impact of $\beta^{*}$ on the home country's quota price is ambiguous. An increase in demand for foreign resource goods encourages firms in the home country's resource goods sector to increase production. While the home country's quota remains unchanged, this will lead to an increase in the home country's quota price. Meanwhile, the increased demand will encourage foreign resource goods sector firms to increase their lobbying efforts. Although foreign firms also increase their production, the foreign quota price will remain unchanged because their quotas as rents are expanded through lobbying. To the extent that the expansion of foreign production corresponds to an increase in foreign demand, firms in the home country's resource goods sector must reduce their supply. Hence, the increase in $\beta^{*}$ leads to a fall in the home country's quota price. As a result of this ambiguous effect of $\beta^{*}$ on the home country's quota price, its taste parameter has a similarly ambiguous effect on labour in the home country's resource good sector. The welfare analysis compares utility functions in autarky economy and free trade economy but does not compare home and foreign utility levels. Therefore, the implications of proposition 3 and proposition 4 in Hamaguchi [12] will not change significantly due to the $\beta \neq \beta^{*}$.

### Method implications

Under an autarkic economy, using (13)–(19), the present method enables us to investigate how changes in parameters related to marine policy $\left(\bar{h},\;f,\;a,\;S,\;T\right)$ and economic structure (β,L) influence welfare, quota price, lobbying effort, employment volume, and production of resource and manufacturing goods. Under an open economy, welfare in the home country changes according to its quota price, while welfare in the foreign country changes according to its own and the home country's quota prices. Additionally, these quota prices depend on parameters related to marine policy $\left(\bar{h},\;{\bar{h}}^{*},\;f,\;f^{*},\;a,\;S,\;T\right)$ and economic structure (β,L). Thus, using (20)–(26), the present method enables us to analyse how changes in parameters related to the marine policy as well as economic structure influence welfare, quota price, lobbying effort, employment volume, and production of resource and manufacturing goods in an open economy. In particular, under an open economy, we can investigate how the lobbying effort (rent-seeking activity) in the foreign country influences welfare through quota prices in both countries. The method is likely to be useful for analysing the relationship between ITQ, rent-seeking, and trade in the international management of fishery resources (see Hamaguchi [12] for more details on the results and implications obtained by comparative statics analysis, as well as limitations and improvements in the present model).

## Declaration of Competing Interest

The authors declare that they have no known competing financial interests or personal relationships that could have appeared to influence the work reported in this paper.
